# Editorial: Recent advancements in neoadjuvant chemotherapy for specific breast cancer subtypes

**DOI:** 10.3389/fonc.2022.1100427

**Published:** 2022-12-13

**Authors:** Sirin A. Adham, Myron R. Szewczuk, Fatima Mraiche, Emanuel Petricoin

**Affiliations:** ^1^Department of Biology, College of Science, Sultan Qaboos University, Muscat, Oman; ^2^Department of Biomedical and Molecular Sciences, Queen’s University, Kingston, ON, Canada; ^3^College of Pharmacy, QU Health, Qatar University, Doha, Qatar; ^4^Institute for Biomedical Innovation, George Mason University, Manassas, VA, United States

**Keywords:** neoadjuvant chemotherapy, TNBC, conservative breast therapy, cardiotoxicity, breast cancer

## Introduction

Most locally advanced breast cancer patients undergo pre-surgery treatment known as neoadjuvant chemotherapy (NAC). The purpose of NAC is to reduce the tumor’s size and improve surgical outcomes, cosmetic results, and chances of conservative breast surgery, control tumor progression and observe tumor sensitivity (or resistance) to the chosen treatment regimen (1–4). Several studies have suggested better survival outcomes in patients achieving complete pathological remission than in patients with residual or progressive disease at the time of definitive surgery (5, 6). However, the mechanisms of primary resistance and strategies to overcome those are a matter of intense research. Triple-negative breast cancer (TNBC) is the most aggressive breast cancer subtype and is responsible for most of the annual mortality rate of breast cancer (7, 8).

This Research Topic focused on studies that tackle the most recent advances in treating breast cancer using NAC. Pegylated liposomal doxorubicin (PLD) is used safely to treat breast cancer patients (9). In addition, it has a superior benefit over free doxorubicin since it is distributed in smaller volumes with extended circulation time (10). A recent clinical trial demonstrated that pegylated liposomal doxorubicin (PLD) is safe for TNBC with a particular focus on elderly patients and those with risks of developing cardiotoxicity (Gil-Gil et al.)

Taxane, carboplatin, and trastuzumab (TCH) neoadjuvant chemotherapy regimen along with HER-2 targeted therapy is the first line treatment for HER-2 positive tumors. However, some patients have residual tumors and do not achieve complete pathological response (pCR) with this regimen (11). Based on the KATHERINE trial, HER-2-positive patients with residual disease are subjected to treatment escalation either by adding another HER-2 inhibitor or prolonging the use of HER-2 adjuvant therapy (12). A study on this topic found that to screen out non-pCR patients, four cycles of TCH are the optimal treatment duration for screening high-risk HER2-positive breast cancer patients for escalation treatment, and there is no need for longer treatment cycles (Xie et al.)

Current cancer treatments focus on tailoring therapies based on unique biomarkers and tumor targets, including immunotherapy. One of the studies on this topic confirmed the efficacy and safety of adding immune checkpoint inhibitors (ICI) to neoadjuvant chemotherapy TNBC, (Li et al.). This study included four different randomized controlled trials. The addition of the ICI to NAC increased the survival of TNBC regardless of the PD-L-1 status of the tumors, which is in line with many other recent studies that showed the positive benefit of adding immunotherapies with traditional NAC for the treatment of early TNBC (13, 14). In support of the latter, Pembrolizumab has been approved by the FDA along with NAC for the treatment of high-risk early-stage TNBC (15).

Following the NAC treatment, most patients would go for surgery. The surgical options can be either mastectomy or oncoplastic breast surgery, the latter usually provides the patient with better quality of life, and it is becoming a more common procedure (16). Breast-conserving therapy (BCT) showed various benefits over mastectomy (17); however, BCT is not considered for patients with tumors in the central and nipple portion (TCNP). The breast tumor location is an independent prognostic factor for breast cancer. Tumors that exist in the upper outer quadrant (UOQ) are the most prevalent and known to have better survival when compared with tumors that arise in the lower inner quadrant (LIQ) and medial regions (18). On the other hand, they had poor prognoses compared to other peripheral quadrants (19). In this topic, the authors added a new hope for patients with TCNP: BCT could be safely used since it exhibited a superior prognosis to mastectomy based on the Surveillance, Epidemiology, and End Results (SEER) database (Wang et al.).

In summary, the topic added recent advancements in NAC for breast cancer, which further contribute to our understanding of the best treatment options. One article concluded that PLD could be used safely in elderly patients, TNBC, and patients prone to cardiovascular diseases. Furthermore, four cycles of the neoadjuvant TCH with anti-HER-2 are the optimum duration for treatment escalation to screen out the patients who don’t achieve pCR. Moreover, the addition of ICI to the neoadjuvant preoperative treatment for TNBC showed better survival regardless of tumor PDL-1 status. Finally, patients with TCNP breast cancer can be operated on using BCT since the results indicated a superior prognosis to mastectomy. The Research Topic added new and valuable information to breast oncologists and clinical researchers.

## Author contributions

All authors listed have made a substantial, direct, and intellectual contribution to the work and approved it for publication.
